# Structure-guided engineering of adenine base editor with minimized RNA off-targeting activity

**DOI:** 10.1038/s41467-021-22519-z

**Published:** 2021-04-16

**Authors:** Jianan Li, Wenxia Yu, Shisheng Huang, Susu Wu, Liping Li, Jiankui Zhou, Yu Cao, Xingxu Huang, Yunbo Qiao

**Affiliations:** 1 School of Life Science and Technology, ShanghaiTech University, Shanghai, China; 2 Precise Genome Engineering Center, School of Life Sciences, Guangzhou University, Guangzhou, China; 3 Department of Orthopaedics and Institute of Precision Medicine, Shanghai Key Laboratory of Orthopaedic Implant Shanghai Ninth People’s Hospital, Shanghai Jiao Tong University School of Medicine, Shanghai, China

**Keywords:** Molecular engineering, CRISPR-Cas9 genome editing

## Abstract

Both adenine base editors (ABEs) and cytosine base editors (CBEs) have been recently revealed to induce transcriptome-wide RNA off-target editing in a guide RNA-independent manner. Here we construct a reporter system containing *E.coli Hokb* gene with a tRNA-like motif for robust detection of RNA editing activities as the optimized ABE, ABEmax, induces highly efficient A-to-I (inosine) editing within an *E.coli* tRNA-like structure. Then, we design mutations to disrupt the potential interaction between TadA and tRNAs in structure-guided principles and find that Arginine 153 (R153) within TadA is essential for deaminating RNAs with core tRNA-like structures. Two ABEmax or mini ABEmax variants (TadA* fused with Cas9n) with deletion of R153 within TadA and/or TadA* (named as del153/del153* and mini del153) are successfully engineered, showing minimized RNA off-targeting, but comparable DNA on-targeting activities. Moreover, R153 deletion in recently reported ABE8e or ABE8s can also largely reduce their RNA off-targeting activities. Taken together, we develop a strategy to generate engineered ABEs (eABEs) with minimized RNA off-targeting activities.

## Abstract (web summary)

Base editors can induce transcriptome-wide RNA off-target editing independent of gRNA. Here, the authors engineer ABEmax variants with minimized RNA off-target activities.

## Introduction

Adenine base editors (ABEs), which is originally designed by fusing a wild-type *E.coli* TadA (ecTadA) and a laboratory-evolved *E.coli* TadA (TadA*) with a Cas9 (D10A) nickase (Cas9n), can induce efficient A-to-G or T-to-C conversions with very low levels of unwanted mutations or insertions^1,2^. ABE is designed based on the native structure of homodimerized ecTadA, which can deaminate an adenosine within a transfer RNA (tRNA)^3^, with an evolved TadA* being capable of deaminating genomic DNA adenosines^1^. Although ABEs show no detectable guide RNA-independent DNA off-target editing^4,5^, both ABEs and cytosine base editors (CBEs) can induce tens of thousands of A-to-I (inosine) or C-to-U (uracil) RNA edits transcriptome-widely in guide RNA-independent manners in human cells^6,7^. Engineered CBE and ABE variants bearing rAPOBEC1 mutations^6^ or TadA/TadA* mutations^7–9^, have been recently reported with reduced RNA off-targeting activities. In these studies^6,8,9^, GATK HaplotypeCaller, a tool for evaluating germline single nucleotide polymorphisms (SNPs) and indels^10^, is employed for analyzing RNA A-to-I edits. It is worth noticing that RNA edits with 0–10% efficiency was not able to be recovered by this tool^6,8,9^, suggesting a possible underestimation of RNA off-targets, therefore, driving us to further examine ABE-induced off-target editing of cellular RNAs in depth. In this work, according to structure-guided principles, we successfully engineered ABEmax and mini ABEmax (TadA* fused with Cas9n) variants to generate engineered ABEs (eABEs) that retained DNA on-target editing activities while largely decreased RNA editing activities.

## Results

### Engineering ABEmax with reduced RNA deamination activities

Considering that cellular RNAs with wide range of aneuploidy copies have been discovered as RNA off-target substrates of ABEs^6–9^, we reasoned that MuTect2, a GATK tool for sensitive detection of somatic point mutations in heterogeneous cancer samples^11^, might be more appropriate than HaplotypeCaller (for detection of euploid germline SNPs) for detection of RNA edits^12^ (Supplementary Fig. 1a). The first step of data analysis with HaplotypeCaller is to identify active regions with high mutation frequency, so the mutated sites with low rates or located in an isolated region might be filtered out in this procedure. Thus, we reanalyzed ABEs- and their optimized variants (mini ABEmax-V82G*, ABE7.10-F148A, and ABEmaxAW)-induced RNA off-targets^6–9^, and found that indeed, MuTect2 recovered 2.7-11-fold number of base editor-induced RNA edits compared with that using HaplotypeCaller, with similar editing signatures (endogenous A-to-I edits were deducted from control sequencing data), demonstrating that these optimized ABE variants still retained a relatively large number of RNA edits (Supplementary Fig. 1b–g). Surprisingly, the overlapped RNA edits from HaplotypeCaller and MuTect2 were as few as 22-68% of HaplotypeCaller-calculated RNA off-targets (Supplementary Fig. 1h–m). We further performed Manhattan plotting for ABEmax-induced RNA off-targets^6^ to show efficiency distributions of overlapped, HaplotypeCaller-specific, and MuTect2-specific RNA edits, respectively, demonstrating that the number of MuTect2-specific RNA edits was much more than HaplotypeCaller-specific edits, especially for those edits with 0–10% editing efficiency, which were ignored by HaplotypeCaller (Supplementary Fig. 1l). Meanwhile, lower overlapping ratio was discovered for the samples possessing fewer RNA edits (Supplementary Fig. 1m). Nine MuTect2-specific edits (with >10% efficiency in RNA-seq data) were randomly selected for PCR validation with cDNAs subjected to RNA-seq experiment. Indeed, all of these amplicons were successfully detected with high or low efficiency of A-to-G mutations (Supplementary Fig. 1n), confirming the reliability of MuTect2-specific edits and suggesting that it is necessary to engineer ABEmax variants based on MuTect2 analysis.

Although an engineered TadA* has been evolved to be capable of deaminating DNA adenines^1,2^, both TadA* and wild-type TadA retain the ability to deaminate cellular RNAs^9^. We analyzed ABEmax-induced RNA edits in ABEmax-overexpressed HEK293T cells from a published RNA-seq dataset (Supplementary Fig. 2a, b)^6^. Meanwhile, we generated our data by co-transfecting HEK293T cells with an sgRNA to efficiently induce DNA A-to-G conversion within ABE site 8 (Supplementary Fig. 2c–e). The cells with highest 15% GFP signal were collected for on-target and off-target analysis (Supplementary Fig. 2f). Higher overlapping ratios between two independent duplicates were observed for those RNA edits with higher editing efficiency, demonstrating the preferential affinity of ABEmax for highly edited RNAs (Supplementary Fig. 2a, d). Thus, we calculated the sequence logos for ABEmax-induced RNA edits with differential scope of editing efficiency, showing that higher-edited adenines preferentially located within a conserved motif being more close to UACGA (Supplementary Fig. 2b, e), which highly resembles the conserved loop region of tRNA substrate for ecTadA^3^. These data demonstrate that, consistent with a recent report^9^, ABEmax induces efficient and transcriptome-wide off-target RNA editing harboring core *E.coli* tRNA-like sequences.

**Fig. 1:**
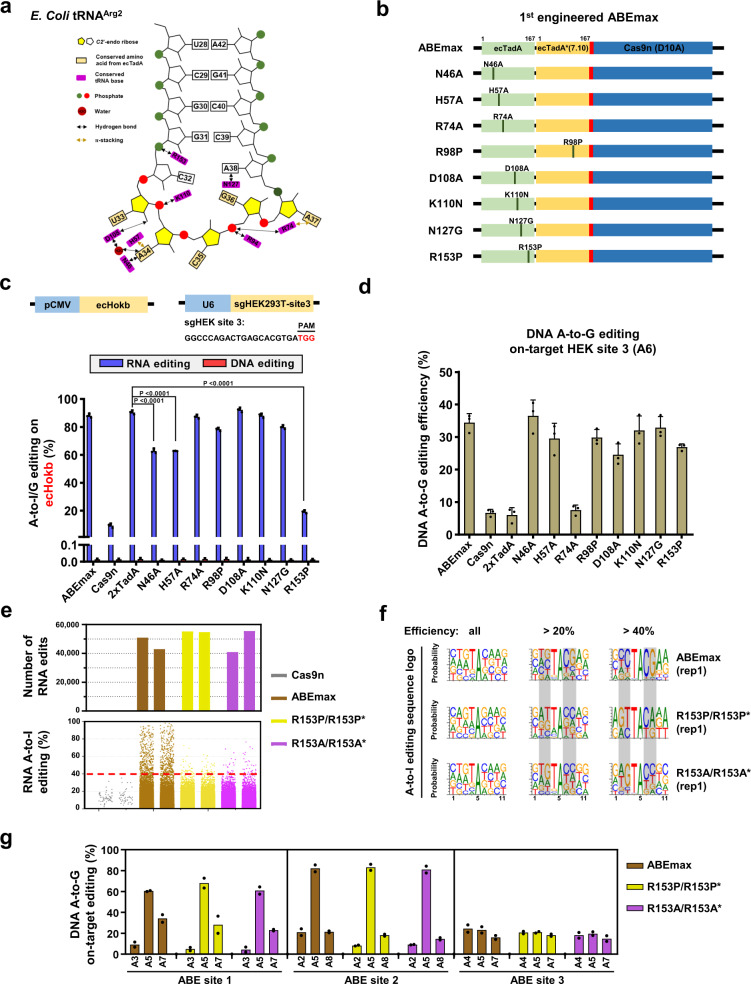
Arginine 153 within TadA/TadA* is essential for specificity of ABEmax-induced RNA deamination. **a** Summary of predicted *E. coli* TadA-tRNA interactions. A classical tRNA^Arg2^ possessing CUACGAA and the potential interaction between the residues from TadA (purple) and tRNA loop nucleotides was shown. **b** Design for the engineered ABEmax (for the 1st round of engineering) with indicated amino acid substitutions. **c** Schematic overview of reporter assays for RNA editing. The upper diagram represents a gene *Hokb* cloned from *E. coli* that is driven by a CMV promoter (pCMV). An sgRNA targeting HEK-site 3 (sgHEK site 3) was constructed into a pGL3-U6 vector. The lower bar plot showed the percentage of A-to-I/G (A-to-G for genomic DNA test; A-to-I for RNA-generated cDNA test) conversion for *Hokb* gene in the reporter, which was co-transfected with sgHEK site 3 and ABEmax variants into HEK293T cells. After transfection for 72 h, cells were harvested for genomic DNA and RNA extraction and reverse transcription, followed by PCR amplification and deep sequencing. A adenine, I inosine, ABEmax codon optimized adenine base editor. Two-tailed *t*-test was performed for comparisons and *P* values were presented. **d** Genomic DNA was subjected to PCR amplification of DNA fragments containing HEK site 3 targeting sites for ABEmax variants, followed by deep sequencing. The A-to-G editing efficiency on adenine 6 (A6) within the sgRNA sequence was shown with triplicates. **e** The number (upper; by MuTect2) and efficiency distribution (lower in Jitter plot) of RNA A-to-I edits were presented for RNA-seq experiments in HEK293T cells that expressed Cas9n (rep1 and rep2), ABEmax (rep1 and rep2), ABEmax R153P/R153P* (rep1 and rep2), or ABEmax R153A/R153A* (rep1 and rep2) and the sgRNA targeting HEK site 8. Two independent replicates were shown. Each dot in the lower panel represents an edited adenine position in RNA. Non-transfected HEK293T transcriptome served as an endogenous editing control, and the RNA A-to-I edits overlapping with endogenous A-to-I edits were excluded. **f** Sequence logos centered by the edited adenine (A) from a single replicate of RNA-seq data in **e** (rep1 for each group) for edited adenines with indicated editing efficiencies (>40%, >20%, and all). The nucleotides around the edited A were shown (nine nucleotides in total). The changed sequence logos for ABEmax variants were highlighted in gray. **g** Bar plot showing the on-target DNA A-to-G editing efficiencies of ABEmax, ABEmax R153P/R153P*, and ABEmax R153A/R153A* with 3 sgRNAs targeting ABE site 1-3. The constructs for these three ABEmax variants were presented in Supplementary Fig. 4c. Data was generated from two independent replicates from deep sequencing analysis. The efficiencies for most highly edited adenines for each sgRNA on-target site within the editing window were reported with two replicates. Error bars represent s.d. Source data are available in the Source Data file.

Therefore, we hypothesized that disruption of the interaction between TadA/TadA* heterodimer and tRNA-loop structure may interfere the catalytic activities of ABEmax on RNA. Since there is no crystal structure information for the complexing between ecTadA and tRNA, we referred the co-crystal structure of *Staphylococcus aureus* TadA (SaTadA) and tRNA as well as the alignment of the conserved amino acid sequences between ecTadA and SaTadA with high similarity^3,13^, showing that the amino acids possibly responsible for interaction with tRNA were conserved between the two types of TadA (Fig. 1a and Supplementary Fig. 3a). Thus, we introduced a series of point mutations into either the TadA or TadA* monomer of ABEmax according to the interacting interface between homodimerized TadA and tRNA^3^ to disrupt TadA/TadA* and tRNA interactions^3^, and measured their RNA and DNA editing activities (Fig. 1b). To facilitate this test, we generated a robust reporter by cloning the *E.coli Hokb* (ecHokb) gene containing tRNA-like CTACGAA sequence, which has been reported to be highly edited by ecTadA at RNA levels^14^, into a CMV promoter-driven vector. Then, this reporter was co-transfected with an sgRNA targeting HEK site 3 and ABEmax or its engineered variants, and the A-to-G editing efficiencies in ecHokb cDNA (reversely transcribed from mRNA) or genomic DNA (gDNA) were determined by targeted deep sequencing on ecHokb cDNA or gDNA amplicons. It showed that both ABEmax and 2xTadA induced highly efficient RNA but not DNA editing within ecHokb locus. Notably, we identified three variants (N46A, H57A, and R153P) with substantially decreased RNA editing activities, especially R153P with most reduced RNA edits comparable to the negative Cas9n control (Fig. 1c and Supplementary Fig. 3b; the endogenous RNA A-to-I edits detected in native HEK293T cells were deducted). In addition, their DNA on-target editing activities were retained (Fig. 1d). Moreover, similar to ABEmax^2^, all variants induced very few by-products and indels (Supplementary Fig. 3c, d). Three amino acids, including N46, R153 (a residue in an α-helix of secondary structure), and the reported site E59^6^, were likely in close contact with tRNA near the enzymatic pocket in structural prediction (Supplementary Fig. 3e). Additionally, ABEmax-R153P variant exhibited comparable DNA on-target A-to-G editing activities for multiple target sites in human cells (HEK293T and U2OS cells) (Supplementary Fig. 4a, b). Thus, we identified three variants, especially ABEmax-R153P, with minimized RNA editing activities in the reporter assay.

Next, we performed RNA-seq analysis to detect transcriptome-wide RNA off-targets induced by ABEmax or R153 substituted variants (R153P, R153P/R153P*, and R153A/R153A*; Supplementary Fig. 4c–e). Surprisingly, the total number of RNA off-targets induced by R153P/R153P* and R153A/R153A* were comparable with ABEmax (Fig. 1e by MuTect2; a bit lower by HaplotypeCaller in Supplementary Fig. 4d), and R153P variant induced even more RNA edits, which was excluded in further test (Supplementary Fig. 4e). In fact, R153P/R153P* and R153A/R153A* was tested after the observation of high RNA A-to-I edits induced by R153P variant. Sequence logos derived from highly edited adenines (> 20%) showed that the preference of ABEmax for a consensus TACG motif was diminished upon R153 substitution (Fig. 1f) and the number of RNA edits with > 40% efficiency for R153P/R153P* and R153A/R153A* was much fewer than ABEmax (Fig. 1e and Supplementary Fig. 4f), suggesting that R153P/R153P* and R153A/R153A* mainly affect highly edited RNA edits harboring a conserved “UACG” motif. These data demonstrate that interfering R153 can minimize the RNA-editing activities of TadA on tRNA loop-like structures, without affecting the DNA on-targeting activity of ABEmax (Fig. 1g).

### Deletion of Arginine 153 (del153) reduces RNA off-targeting activities in engineered ABEs

**Fig. 2:**
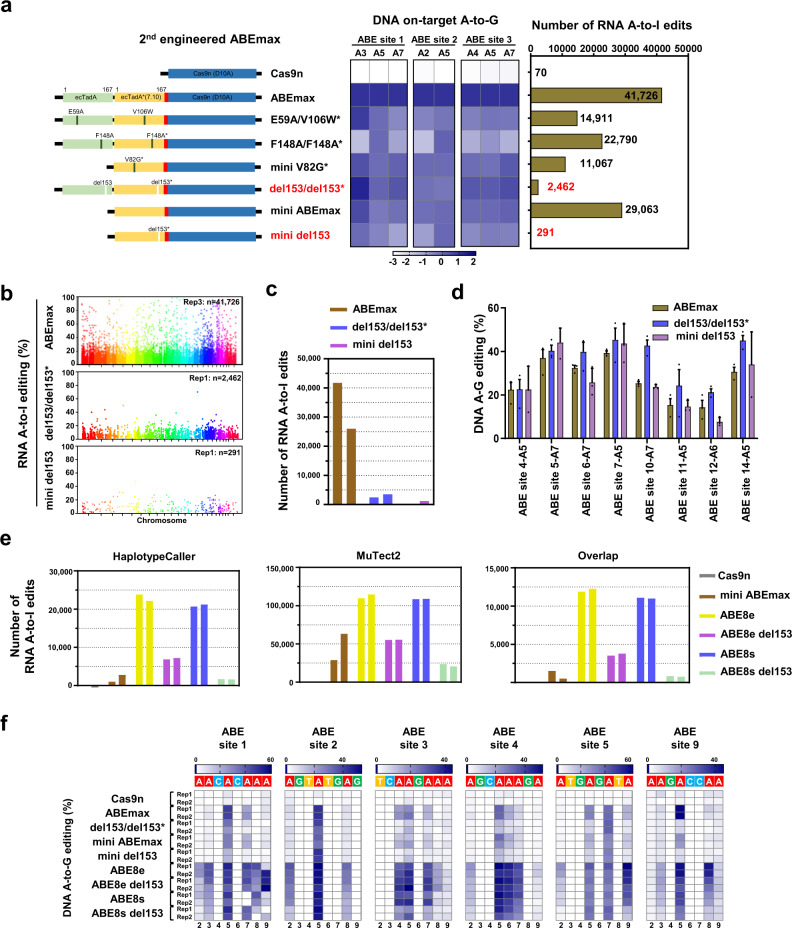
Deletion of R153 from TadA/TadA* results in minimized RNA off-targeting activities. **a** Left panel: design for the second round of engineered ABEmax variants with R153 deletions (del). Asterisk represents amino acid substitutions or deletions constructed in TadA*. mini ABEmax was fused by TadA* and Cas9n. R153 was deleted from TadA* to generate mini del153. Middle panel: heat maps showing DNA on-target A-to-G editing efficiency in HEK293T cells co-expressing ABEmax or its variants (as shown in left panel) with sgRNAs targeting ABE site 1, 2, and 3, respectively. Efficiencies were presented in heat map as log_2_(fold change) for each site; each box representing the DNA A-to-G editing efficiency normalized to the that observed with ABEmax for each on-target DNA sites (ABE site 1, 2, and 3). The efficiencies for edited adenines within the window (4-8 of the protospacers) of the on-target sites for these three sites were shown. Right panel: the number of RNA A-to-I edits (calculated by MuTect2) induced by engineered ABEmax variants was presented; ABEmax (rep3) and Cas9n (rep3) served as controls. Non-transfected HEK293T transcriptome served as a control, and the RNA A-to-I edits overlapping with endogenous A-to-I edits were excluded. **b** Manhattan plots showing the representative distributions of RNA A-to-I edits across all chromosomes induced by ABEmax (rep3), del153/dele153* (rep1), and mini del153 (rep1) with an sgRNA targeting ABE site 8 in HEK293T cells from RNA-seq data. **c** The diagram showing the number of RNA A-to-I edits induced by ABEmax (rep3 and rep4), del153/dele153* (rep1 and rep2), and mini del153 (rep1 and rep2) with an sgRNA targeting ABE site 8 in HEK293T cells from RNA-seq analysis. **d** Bar plots showing the DNA on-target A-to-G editing efficiencies of ABEmax, del153/dele153*, and mini del153 with 8 sgRNAs. The efficiencies for most highly edited adenines for each sgRNA on-target site within the editing window are reported, and error bars represent mean ± s.d. of three independent experiments. **e** The number of RNA edits calculated by HaplotypeCaller or MuTect2, or overlapped RNA edits (HaplotypeCaller and MuTect2) was presented for RNA-seq experiments in HEK293T cells that expressed Cas9n, mini ABEmax, ABE8e, ABE8e del153, ABE8s, ABE8s del153, and an sgRNA targeting HEK site 8. Two independent replicates were shown. **f** Heat maps showing DNA on-target A-to-G editing efficiencies of Cas9n, ABEmax, del153/dele153*, mini ABEmax, mini del153, ABE8e, ABE8e del153, ABE8s, and ABE8s del153 with six sgRNAs. Data are generated from two independent replicates. A-to-G editing efficiencies are shown in heat map format for the editing window 2–9. Numbering at the bottom of the heat map represents spacer position and “1” was calculated from the most PAM-distal nucleotide. Source data are available in the Source Data file.

R153 was essential for deamination activity of ABEmax on conserved tRNA loop-like structures (Fig. 1), while mutation of R153 did not reduce the total number of RNA edits, suggesting that R153 substitution cannot fully disrupt its RNA affinity. Deletion of essential amino acid has been reported as an important strategy to change protein conformation and substrate affinity for protein engineering^15–17^, so we tried to engineer ABEs with reduced RNA off-targets by deleting R153 from both TadA and/or TadA* within ABEmax (del153/del153*) or mini ABEmax^9^ (mini del153). As expected, we demonstrated that compared with ABEmax or mini ABEmax, the RNA off-targets induced by del153/del153* and mini del153 were largely decreased, and there were as few as 291 (MuTect2) or 98 (HaplotypeCaller) RNA A-to-I edits for mini del153 group (Fig. 2a–c and Supplementary Fig. 5a), while both variants retained a relatively high DNA on-targeting activity (Fig. 2a). We then overlapped or merged the ABEmax-, del153/del153*-, or mini del153-induced RNA A-to-I edits using HaplotypeCaller and MuTect2, respectively. Compared with ABEmax, both del153/del153* and mini del153 induced remarkably decreased RNA edits of the overlapped, HaplotypeCaller-specific, MuTect2-specific, and merged edits (Supplementary Fig. 5b). Manhattan plots and histograms further confirmed that both the number and efficiency for del153/del153*- and mini del153-induced RNA A-to-I edits were strikingly decreased (Fig. 2b, c), accompanying with much lower mean frequencies throughout the transcriptome (Supplementary Fig. 5c).

Next, we compared our engineered ABE variants (eABEs) with reported variants possessing reduced RNA off-targeting activities, including ABEmaxAW (E59A/V106W*)^7^, ABE-F148A/F148A*^8^, and SECURE-ABEs (mini ABEmax-V82G*)^9^. The results showed that our eABE variants, del153/del153* and mini del153, induced much fewer RNA off-targets by both MuTect2 and HaplotypeCaller tools under the same experimental conditions (with much fewer RNA edits by using HaplotypeCaller; Fig. 2a and Supplementary Fig. 5a).

We further characterized the DNA on-target editing activities of del153/del153* and mini del153 variants for another eight target sites. It demonstrated that the DNA on-targeting activities of del153/del153* and mini del153 were similar to ABEmax for nearly all of detected sites, except for ABE site 12 with a bit lower but satisfactory editing efficiency (Fig. 2d and Supplementary Fig. 6a). Similar to SECURE-BE3-induced RNA C-to-U edits with perfect reducing effect^6^, del153/del153* and mini del153 variants-induced RNA A-to-I edits were decreased to only dozens or hundreds of off-targets when using HaplotypeCaller (Supplementary Fig. 5a). Comprehensively considering the on-targeting and off-targeting activities of engineered variants, we designate del153/del153* and mini del153 as our best optimized ABE variants with minimized RNA editing activities. Recently reported ABE8e and ABE8s containing evolved mutations within TadA/TadA* possess increased DNA on-targeting activities as well as elevated RNA off-targeting activities^18,19^. We tried to further engineer ABEs with higher DNA on-targeting and lower RNA off-targeting activity by deletion of R153 from ABE8e or ABE8s, demonstrating that RNA off-targets were also remarkably decreased from HaplotypeCaller or MuTect2 calculations, and the number of ABE8s del153 exhibited comparable number of RNA edits with mini ABEmax (Fig. 2e and Supplementary Fig. 6b). Notably, ABE8e del153 and ABE8s del153 showed comparable or slight lower levels of DNA A-to-G editing activities than ABE8e/8s; however, the on-targeting activity of ABE8e/8s or ABE8e/8s del153 was much higher than ABEmax or mini ABEmax. Moreover, the editing window of ABE8e/8s or ABE8e/8s del153 was much wider than ABEmax or mini ABEmax (Fig. 2f and Supplementary Fig. 6c), and all adenines within the window 3-9 were highly edited by ABE8e/8s or ABE8e/8s del153. Lastly, we compared the DNA A-to-G editing activities of all constructed ABE variants, and R153 deletion or mutation (R-to-A/P/E) showed none or tolerable reducing effects on their on-targeting activities (Supplementary Fig. 7a). Meanwhile, low levels of unexpected A-to-nonG conversion rates and indel rates were observed, with a little higher indel rates for ABE8e and ABE8e del153 (Supplementary Fig. 7b, c). In collection, we propose that deletion of R153 is a feasible strategy for reducing RNA off-targeting activities in engineered ABEs.

## Discussion

Our description of ABEmax-induced transcriptome-wide RNA off-targeting with high frequency and efficiency confirmed the findings in recent studies^7–9^, although the number of RNA A-to-I edits was variable, possibly because of differential expression of ABEs^8^ (Supplementary Fig. 6) and detection methods. When we noticed the rare distribution of RNA edits with 0-10% efficiency using HaplotypeCaller^6,8,9^, we started using MuTect2, a widely used tool for calling somatic mutations in cancers^11,20^, which might be more suitable for analyzing SNPs within aneuploid mRNAs. Surprisingly, we identified 2.7-11-fold number of ABEs-induced RNA edits, only 22–68% of which was overlapped with HaplotypeCaller-generated edits (Supplementary Fig. 1). We conclude that the number of BEs-induced RNA off-targets is underscored, especially for those edits with <10% editing efficiency, which may result in some poisoned or oncogenic proteins in therapeutic cases^21^. The sequence logos analysis suggests that TadA/TadA* preferentially edit cellular RNAs with an “UACGA” motif, not regarding the secondary structure of RNAs. Detection performances of different tools to call RNA mutations highly depend on the sequencing depths, detected regions, and variant allele frequencies^12^, which may lead to differential results from different tools. Therefore, barely using MuTect2 is not the best way to examine RNA A-to-I edits, and developing a new tool by computational scientists, such as combining HaplotypeCaller and MuTect2^11,22^, for more accurate evaluation of RNA off-targeting effect will be quite helpful^23^.

Based on a structure-guided design^3^ to disrupt the interaction between TadA/TadA* and tRNA-like mRNAs with conserved UACGA motif^9^, we successfully identify R153 as an important amino acid for deaminase activity of TadA/TadA*, supported by R153A/R153A* variants-induced lower efficiency of RNA A-to-I edits. Interestingly, the RNA editing efficiency for our reporter ecHokb and those efficiently edited RNAs with tRNA loop-like structures by ABEmax was markedly decreased upon R153 substitution, whereas the total number of RNA edits was not significantly changed (Fig. 1). It indicates that R153 might be required for TadA to specifically bind to t-RNA loop-like RNAs, and deletion of R153 within TadA/TadA* in del153/del153* and mini del153 variants strikingly reduces the number of RNA off-targets, with high DNA on-targeting activity retained (Fig. 2), further confirming the reasonability of our strategy. However, mutation of R153 into “P” or “A” may retain its structural interaction with RNAs, while deletion of R153 or mutation into an acidic amino acid “E” may disrupt this interaction, because R153E (“positive” to “negative” charge) mutant displayed a little lower on-targeting activity as del153/del153* (Supplementary Fig. 7). We propose that deletion of R153 can largely decrease the deamination activities of ABEmax and mini ABEmax. Moreover, our del153/del153* and mini del153 variants show better optimizing effects than the reported versions under our experimental conditions. When comparing with the perfect reducing effect of SUCURE-BE3-induced RNA C-to-U edits^6^, del153/del153* and mini del153 variants-induced RNA A-to-I edits are decreased to only dozens or hundreds of off-targets when using HaplotypeCaller. Considering a slightly lower DNA on-targeting efficiency for mini del153 occasionally, del153/del153* is priorly recommended for targets with low targeting efficiencies.

We also combine del153 strategy with evolved ABEs, ABE8e and ABE8s^18,19^, demonstrate that deletion of R153, the residue nearby some of mutated acids in ABE8e/ABE8s^18,19^, can also remarkably reduce the number of RNA edits but retain their on-targeting activities in most cases (Fig. 2). Therefore, ABE8e del153 and ABE8s del153 are suitable for desiring higher DNA on-targeting and lower RNA off-targeting activities. Besides, it has been reported that BEs-induced RNA off-target editing acts in an sgRNA-independent manner^6,9^, thus we do not consider the sgRNA-dependent effects in the current study. While these findings remind us to reconsider the off-targeting activities of our and others’ reported dCas9-fused epigenome editing tools^24,25^. In addition, replacement of the deaminases or Cas9n of base editors, such as APOBEC3B, APOBEC3G, or YE1 variants, is a feasible strategy to reduce their sgRNA-independent DNA or RNA off-targeting activities^26–28^.

In sum, we reveal R153 of TadA/TadA* as an essential amino acid for its RNA deamination ability, and we successfully optimize ABEs by deletion of R153 from TadA/TadA* to generate eABEs, which greatly reduce the number of RNA edits while retain high DNA on-targeting activities. The successful engineering of CBEs and ABEs variants in our and other two studies^6–9^ expands our understanding of desired and undesired features of DNA and RNA editing activities of base editors, and provides a feasible pathway available to engineer base editors based on structure-guided design to minimize the unwanted properties while retaining the desired on-targeting ability for CBEs and ABEs.

## Methods

### Plasmid construction

Briefly, the synthesized DNA oligos for sgRNA-expressing plasmid construction were annealed and cloned into pGL3-U6-sgRNA-PGK-EGFP with U6 promoter (Addgene #107721). Oligos are showed in Supplementary Table 1. Base editors were constructed by insertion of amplified DNA product into linearized ABEmax (NdeI/Bg1II digest of pCMV_ABEmax, Addgene #112095). DNA products were amplified by Phanta Max Super-Fidelity DNA Polymerase (Vazyme, P505) using mutant site-containing primers (such as TadA-N46A-F/R) and two fragments primer sets (CMX-NdeI-F and Cas9n-Bg1II-R). Primers used are shown in Supplementary Table 2.

### Cell culture and transfection

HEK293T and U2OS cells were purchased from ATCC and cultured in DMEM (10566, Gibco/Thermo Fisher Scientific) supplemented with 10% fetal bovine serum (FBS) (v/v) (Gemini, 900-108) and 1% Penicillin Streptomycin at 37 °C with 5% CO2.

For deep sequencing samples, HEK293T cells were seeded on 24-well plates (JETBIOFIL) and transfected at ~70% confluence with editors (628 ng) and sgRNAs (373 ng) using Lipofectamine LTX (ThermoFisher Scientific, 15338100) according to the manufacturer’s protocol. GFP positive cells were harvested from fluorescence-activated cell sorting (FACS) 48 h after transfection.

For RNA sequencing samples, HEK293T cells were seeded on 6 cm dish (JETBIOFIL) and transfected at ~70% confluence with editors (4 µg) and sgRNA-expressing plasmids (2 µg) using Lipofectamine LTX (ThermoFisher Scientific, 15338100) according to the manufacturer’s protocol. GFP signal positive cells of top 15% were harvested from fluorescence-activated cell sorting (FACS) 48 h after transfection.

### RNA and genomic DNA extraction

Genomic DNA of HEK293T and U2OS cells was extracted using phenol-chloroform method. For RNA extraction, cells harvested from FACS were immediately treated with TRIzol reagent (Vazyme, R401-01), according to the manufacturer’s instructions.

### Targeted deep sequencing

Target sites were amplified with primers listed in Supplementary Table 3 using Phanta® Max Super-Fidelity DNA Polymerase (Vazyme, P505). PCR products with different barcodes were pooled together for deep sequencing on Illumina Nextseq 500 (2 × 150 PE) platform at the Novogene Bioinformatics Institute, Beijing, China. BWA (V0.7.16) and Samtools (V1.9) was employed for mapping the pair-end reads to human reference genome (hg38). The adapter pair of the pair-end reads were removed using AdapterRemoval version 2.2.2, and pair-end read alignments of 11 bp or more bases were combined into a single consensus read. All processed reads were then mapped to the target sequences using the BWA-MEM algorithm (BWA V0.7.16). For each site, the mutation rate was calculated using bam-readcount with parameters -q 20 -b 30. Indels were calculated based on reads containing at least 1 inserted or deleted nucleotide in protospacer. Indel frequency was calculated as the number of indel-containing reads/total mapped reads.

### RNA off-target analysis by RNA-seq

The libraries were sequenced on an Illumina HiseqXten-PE150, at a depth of ~20 million reads per sample. The reads were mapped to the human reference genome (hg38) by STAR software (Version 2.5.1); annotation from GENCODE version V30 was used. After removing duplication, variants were identified by GATK (Version 4.1.8.1; MuTect2 and HaplotypeCaller). For MuTect2 method, variants were filtered with FilterMutectCalls. For HaplotypeCaller method, variants were first filtered with QD (Quality by Depth) <2, then all variants were verified and quantified by bam-readcount with parameters -q 20 -b 30. The depth for a given edit should be at least 10x and these edits were required to have at least 99% of reads supporting the reference allele in the wild-type samples. Finally, only A-to-G edits in transcribed strand were considered for subsequent analysis. Motif or sequence logo was analyzed by WebLogo (v3.6.0) for RNA edits. The downloaded data subjected to RNA off-target analysis from four published papers were listed in Source Data for Supplementary Figures. Detailed information for called mutations was provided in in a Source Data file (Source Data for called mutations from RNA-seq data).

### Structural analysis

A structural model for TadA-RNA complex was generated using coordinates from PDB ID 2B3J (weblink: http://www.rcsb.org/structure/2b3j) by PyMol (The PyMOL Molecular Graphics System, Version 1.9 Schrödinger, LLC.). TadA from *Staphylococcus aureus* (SaTadA) was shown as cartoon model in gray and the RNA bound was shown as stick model rendered by elements, with the Zn^2+^ ion as green sphere. The residues critical for the RNA binding of TadA were shown in ball-and-stick model and labeled with single-letter codes in red.

### Statistics

Results were obtained from two or three independent experiments and were presented as the mean ± s.d. All original data presented in main figures were provided in a Source Data file (Source Data for Main Figures), and original data presented in supplementary figures were presented in a Source Data file (Source Data for Supplementary Figures). Statistical analyses and graphing were carried out by using GraphPad Prism 8.0. Comparisons of mean values were analyzed by Student’s *t* test.

### Reporting summary

Further information on research design is available in the Nature Research Reporting Summary linked to this article.

## Supplementary information

Supplementary Information

Reporting Summary

## Data Availability

All sequencing data was provided in SRA (accession number: PRJNA660634). All relevant data are available from the authors. Source data are provided with this paper.

## Source data

Source Data
