# Supplementary material for: Structure-guided engineering of adenine base editor with minimized RNA off-targeting activity
Source: Nat Commun. 2021 Apr 16;12:2287. doi: 10.1038/s41467-021-22519-z (PMC8052359; doi:10.1038/s41467-021-22519-z)
Supplement: Supplementary file 1 — Supplementary Information [file 41467_2021_22519_MOESM1_ESM.pdf]

a

Comparisons \ Tools	HaplotypeCaller	MuTect2
Function	Call germline SNPs and indels	Call somatic SNPs and indels
Reference	Reference genome	Normal control
Need to identify active regions	Yes	No
Ploidy	Fixed ploidy	Multiple ploidy

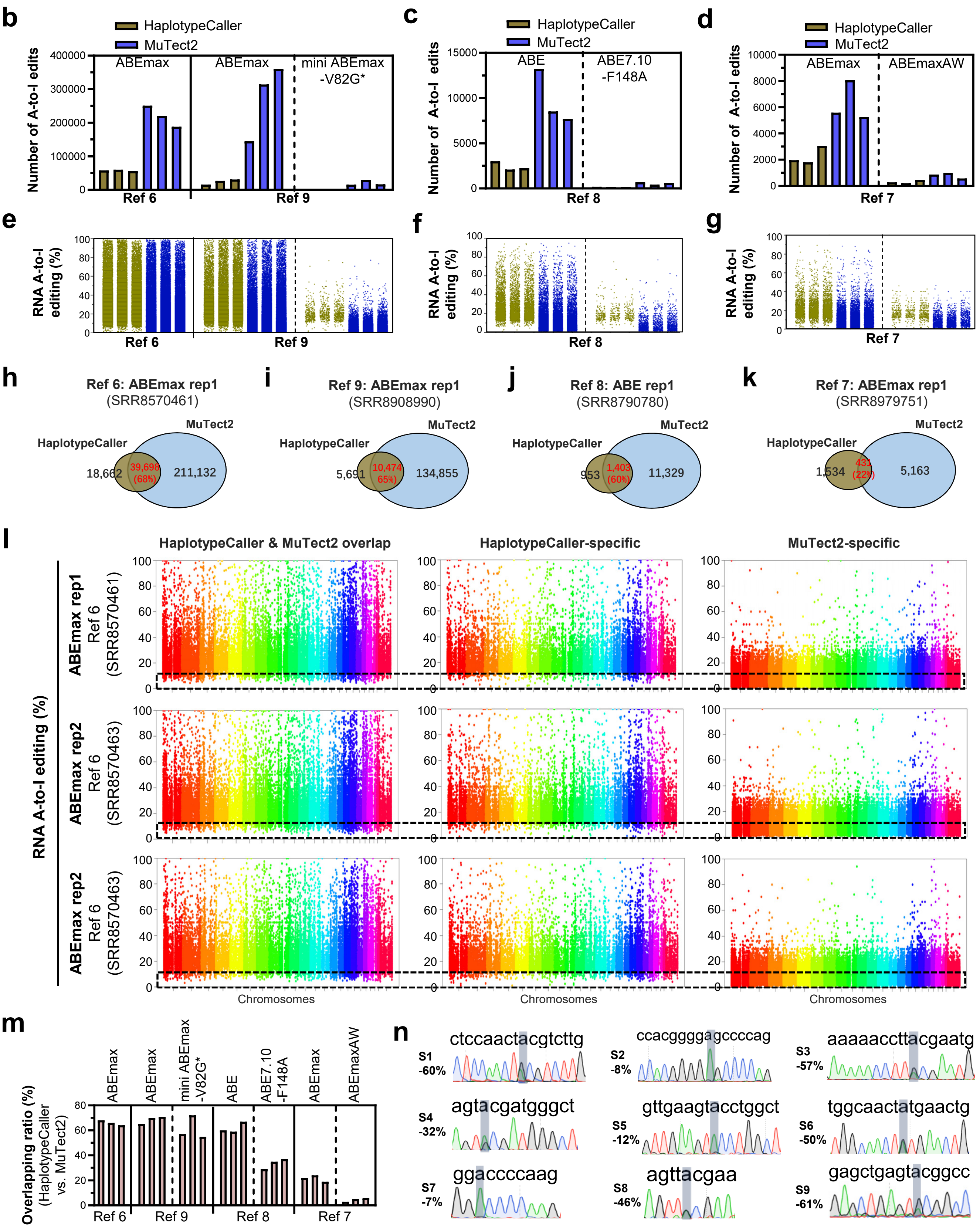

Figure S1. ABE-induced RNA off-target editing analysis.

Figure S1. ABE-induced RNA off-target editing analysis.

(a) Comparison of two widely used tools (HaplotypeCaller and MuTect2) for calculating SNPs.

(b-d) Calculating ABE-induced RNA off-target editing in four published papers (Ref 6-9; the detailed dataset used was listed in a Source Data file (Source Data for Sup Figures) using HaplotypeCaller and MuTect2 separately. Because there was no sequencing data available for non-transfected samples, samples expressing control GFP or Cas9n served as controls, and the RNA A-to-I edits overlapping with control were deducted for calculation.

(e-g) Jitter plots showing the efficiencies of RNA A-to-I edits from the RNA-seq data shown in b-d.

(h-k) The RNA A-to-I edits calculated by HaplotypeCaller and MuTect2 were overlapped for indicated dataset, and the number of overlapped edits and the overlapping ratio relative to HaplotypeCaller-generated edits were shown as disproportional Venn diagram for four published RNA-seq data (SRR8570461, SRR8908990, SRR8790780, and SRR8979751) (Ref 6-9).

(l) Manhattan plots showing the overlapping or exclusive distributions of RNA A-to-I edits across all chromosomes induced by ABEmax from published RNA-seq data (SRR8570461, SRR8570463, and SRR8570465) using HaplotypeCaller and MuTect2 tools. The black rectangle highlights the main difference between the results from HaplotypeCaller and MuTect2.

(m) The overlapping ratio for RNA A-to-I edits calculated by using HaplotypeCaller (as denominator) and MuTect2 tools was shown from the results shown in b-d.

(n) 9 MuTect2-specific RNA edits (ABE-MU-OFT-1 to ABE-MU-OFT-8; also shown as S1-S8) were selected for PCR validation (from cDNA) and Sanger sequencing. The A-to-G conversion efficiency was also presented. The primers for amplifying the fragments containing these edits are listed in Supplementary Table 3.

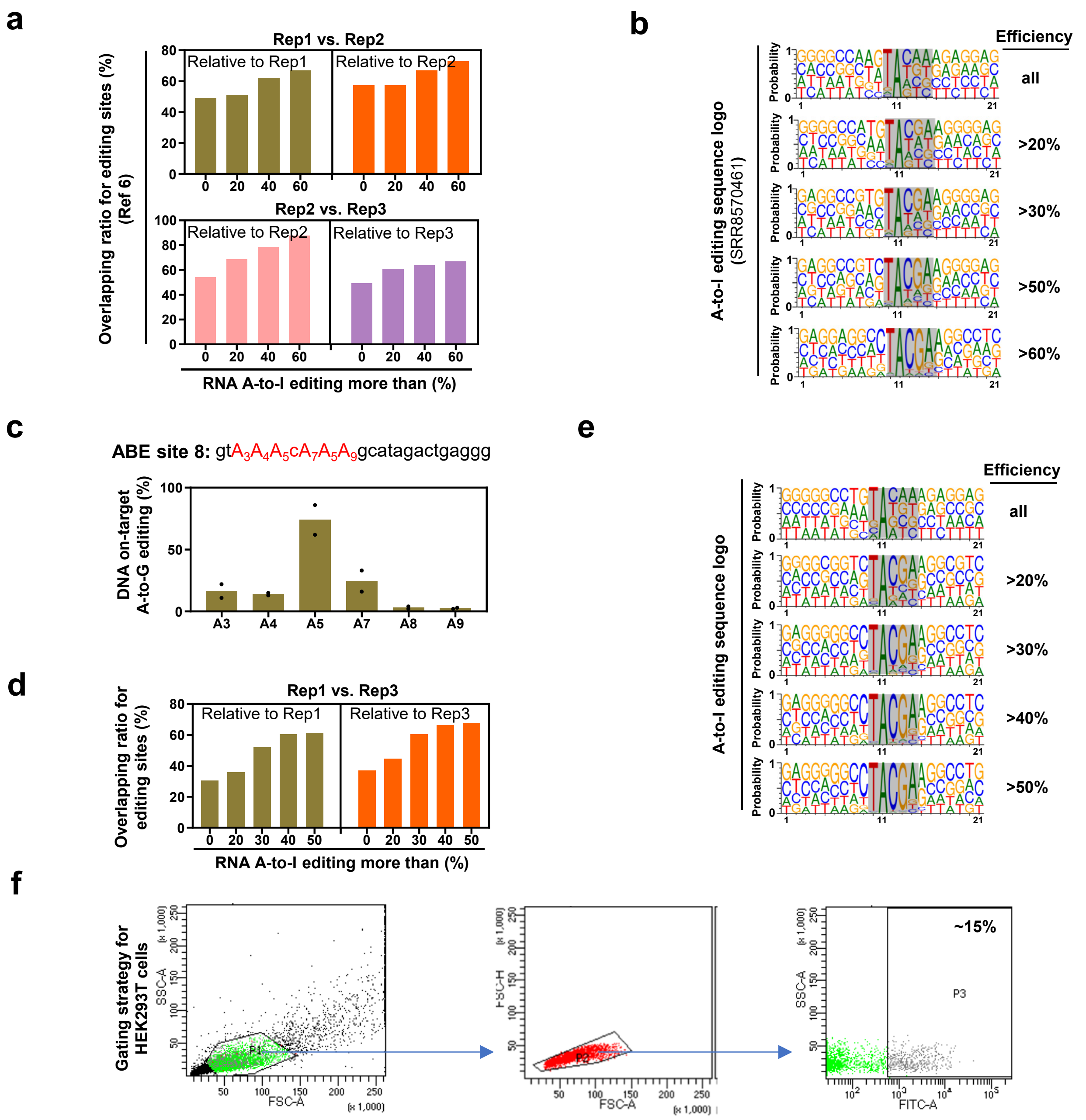

Figure S2. ABEmax induces efficient transcriptome-wide RNA editing with *E.coli* tRNA-like structure.

Figure S2. ABEmax induces efficient transcriptome-wide RNA editing with *E.coli* tRNA-like structure.

(a) The overlapping ratio between the three replicates (rep1, 2, and 3) (Ref 6) divided into four groups of edited adenines edits using MuTect2 (with RNA editing efficiency > 60%, > 40%, > 20%, and all).

(b) Sequence logos centered by the edited adenine (A) from a published RNA-seq data (SRR8570461 from Ref 6) for edited adenines with indicated editing efficiencies (> 60%, > 50%, > 30%, > 20%, and all).

(c) HEK293T cells were transfected with ABEmax and an sgRNA targeting ABE site 8. The DNA on-target A-to-G editing efficiency with two independent replicates was shown from Sanger sequencing data.

(d) Two replicates (ABEmax rep1 and rep3) in c were collected for RNA-seq analysis. The overlapping ratio between the two replicates divided into five groups of edits using MuTect2 (with RNA editing efficiency > 50%, > 40%, > 30%, > 20%, and all).

(e) Sequence logos centered by the edited adenine (A) from pooled RNA-seq data in d for edited adenines with indicated editing efficiencies (> 50%, > 40%, > 30%, > 20%, and all). The nucleotides around the edited A were shown (21 nucleotides in total).

(f) Gating strategy for sorting GFP-positive HEK293T cells was presented separately.

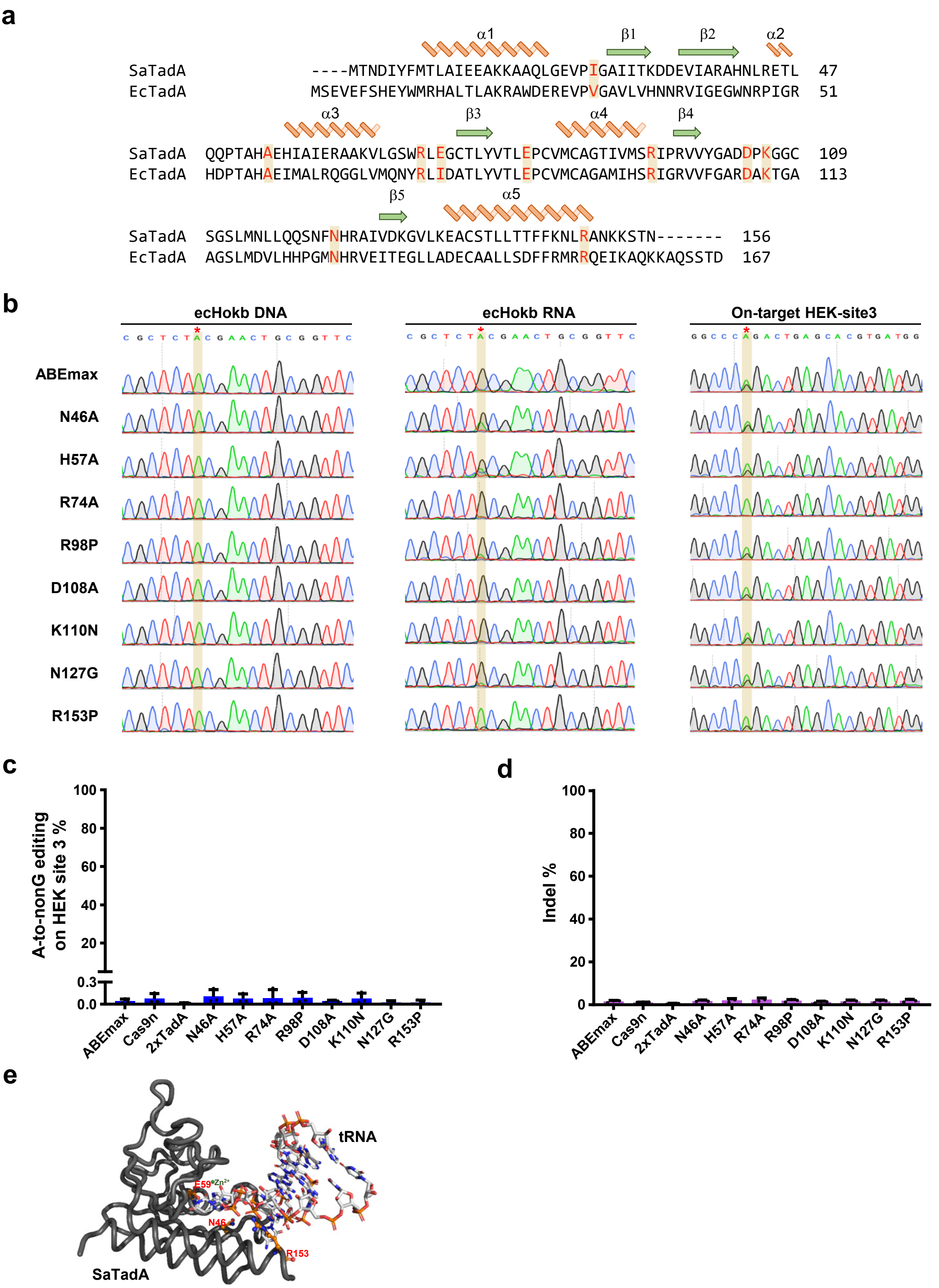

Figure S3. Additional data for engineered ABEmax with reduced RNA editing activities.

Figure S3. Additional data for engineered ABEmax with reduced RNA editing activities.

(a) Protein sequence alignment of *Staphylococcus aureus* TadA (SaTadA) and *E. coli* TadA. The conserved essential amino acids were highlighted in red. The secondary structure of TadA was also labelled. α, α-helix; β, β-sheet.

(b) Sanger sequencing for DNA A-to-G editing and RNA A-to-I editing of ecHokb induced by wild-type ABEmax and eight variants containing indicated mutations. The diagram for on-target DNA editing of HEK site 3 was also presented.

(c-d) Deep sequencing analysis of the fraction of by-products, including A-to-nonG editing (c) and indels (d), induced by wildtype ABEmax and engineered variants. Three independent replicates are presented as mean values ± s.d..

(e) The predicted crystal structure presenting the interaction between SaTadA and tRNA. The contact sites between N46, E59, and R153 of ecTadA and tRNA were highlighted.

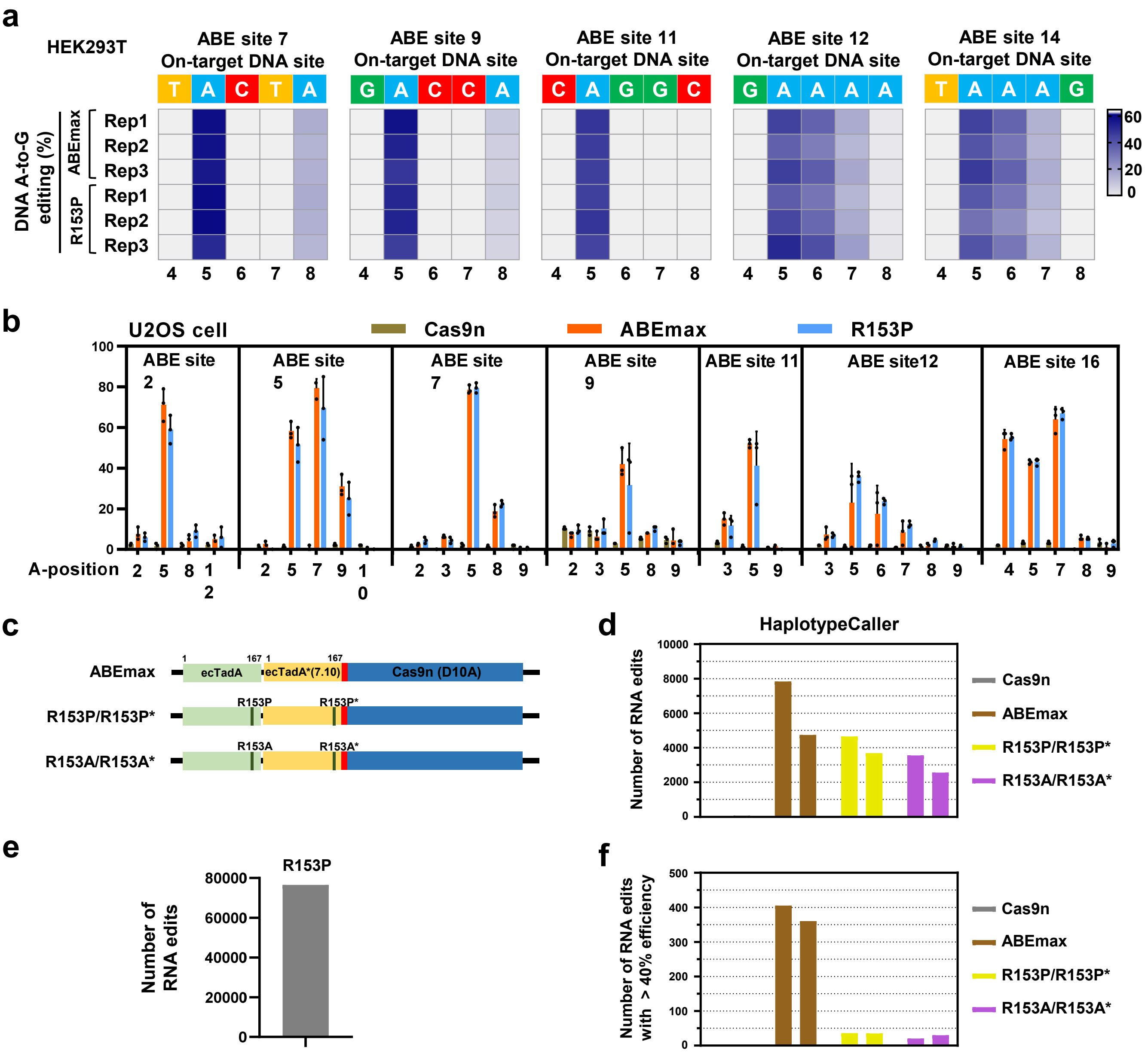

Figure S4. The DNA A-to-G editing efficiency for ABEmax variants.

Figure S4. The DNA A-to-G editing efficiency for ABEmax variants.

(a) Heatmaps showing the DNA on-target editing efficiencies induced by ABEmax or its variant ABEmax-R153P within the editing window (4-8 of the protospacers) of the on-target sites (ABE site 7, 9, 11, 12, and 14) in HEK293T cells by deep sequencing analysis.

(b) The on-target DNA editing events induced by ABEmax or ABEmax-R153P variant within the editing window of the seven on-target sites (ABE site 2, 5, 7, 9, 11, 12, and 16) were presented in U2OS cells by Sanger sequencing analysis in triplicates. Cas9n served as a negative control. Because the transfection efficiency for U2OS cells is very low, we isolated gDNA and RNA from FACS-sorted cells with the highest 5% of GFP signal (gating strategy was similar to HEK293T cells and top 5% GFP-positive cells were collected for analysis).

(c) Design for the engineered ABEmax variants with indicated amino acid substitutions.

(d) The number of RNA A-to-I edits by using HaplotypeCaller in Fig. 2e was presented.

(e) The number of ABEmax-R153P-induced RNA A-to-I edits (MuTect2) with one replicate was presented, which is simultaneously generated with the data in Fig. 2e.

(f) The number of RNA A-to-I edits with > 40% editing efficiency (MuTect2) in Fig. 2e was presented.

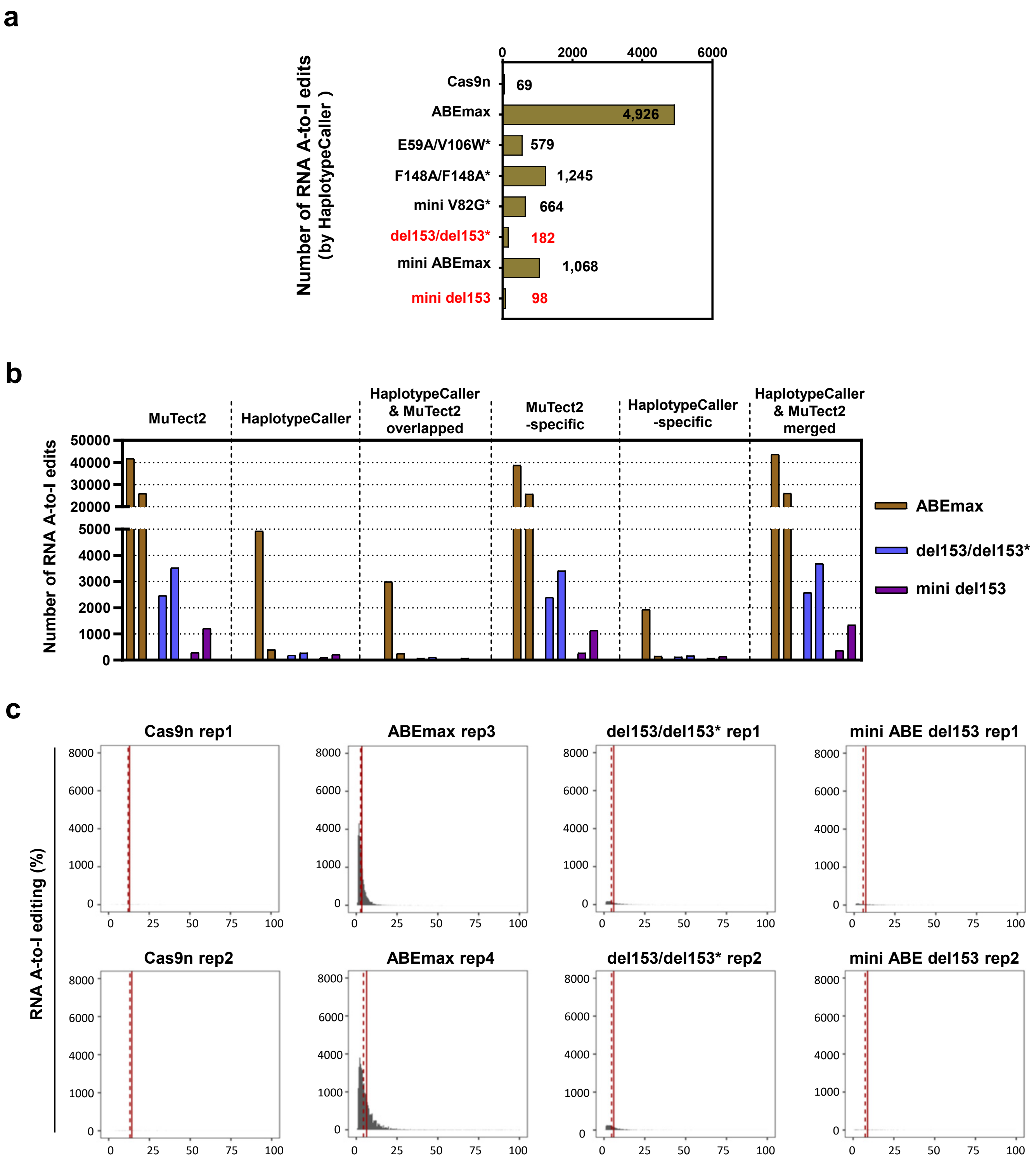

Figure S5. Additional analysis for engineered ABEmax-induced RNA off-targeting activities.

Figure S5. Additional analysis for engineered ABEmax-induced RNA off-targeting activities.

(a) The number of RNA A-to-I edits by using HaplotypeCaller in Fig. 2a was presented.

(b) The RNA A-to-I edits induced by ABEmax or its variants (del153/dele153* and mini del153) expressed in HEK293T cells co-transfected with an sgRNA targeting ABE site 8 were calculated by using HaplotypeCaller and MuTect2 tools separately. Then, the RNA A-to-I edits generated by the two tools were overlapped for each sample, and the number of overlapped edits, HaplotypeCaller-/MuTect2-specific edits, and merged edits was shown. ABEmax, rep 3 and rep4; del153/dele153*, rep1 and rep2; mini del153: rep1 and rep2. Two independent replicates are presented separately.

(c) Histograms showing numbers of off-target edited RNA adenines (y-axis) and corresponding RNA A-to-I editing frequencies (x-axis) for two replicates shown in Fig. 2b (using MuTect2). Solid red line represents the mean frequency, and dashed red line represents the median frequency.

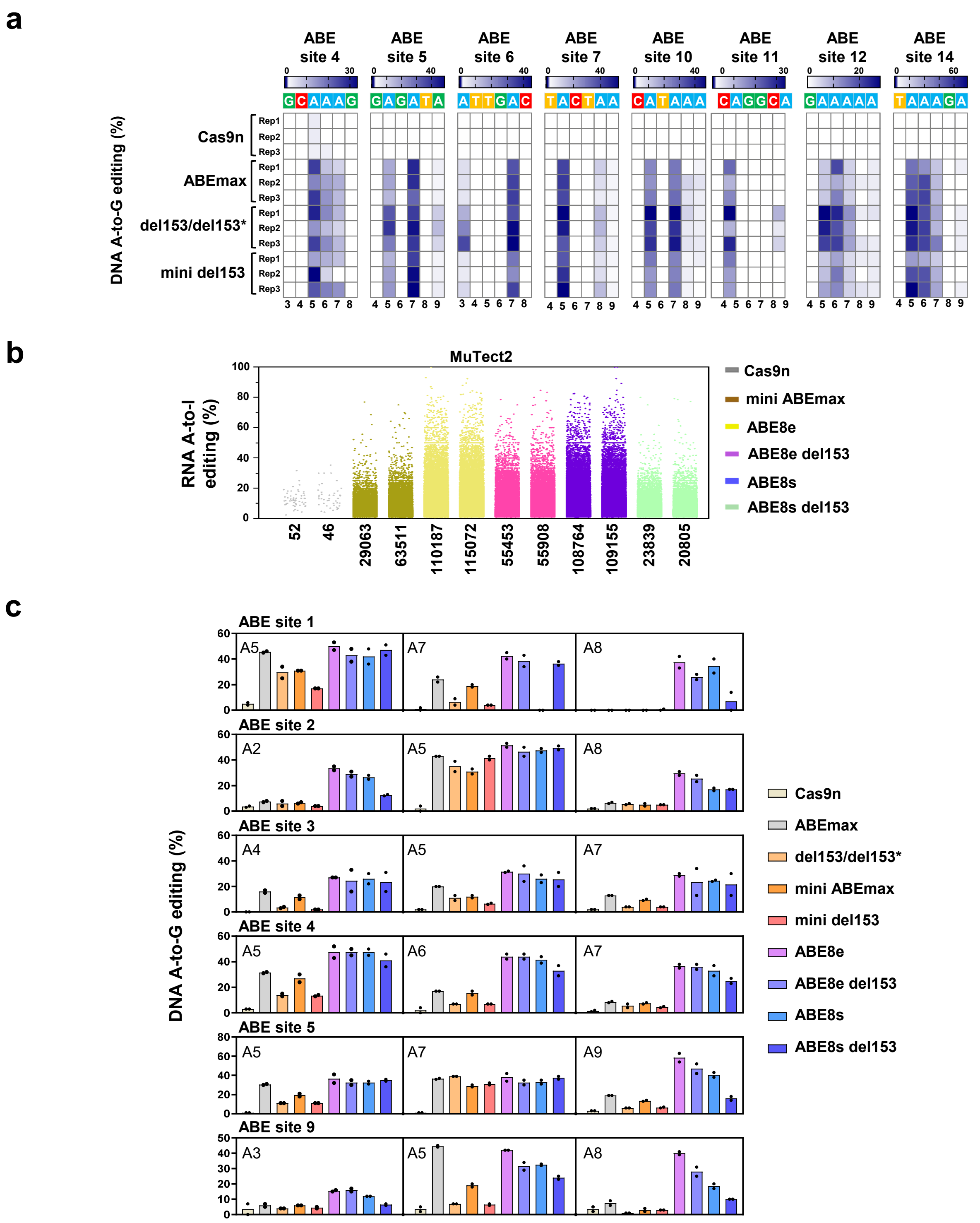

Figure S6. The DNA on-target and RNA off-target activities of engineered ABEs.

Figure S6. The DNA on-target and RNA off-target activities of engineered ABEs.

(a) Heat maps showing the on-target DNA A-to-G editing efficiencies of ABEmax, del153/dele153* and mini del153 with 8 sgRNAs. Data are produced from two independent replicates. A-to-G editing efficiencies are shown in heat map format for the editing window that only includes the most highly edited adenines. Site 4-6, deep sequencing; site 7, 10, 11, 12, 14, Sanger sequencing. Numbering at the bottom of the heat map represents spacer position and "1" is calculated from the most PAM-distal nucleotide.

(b) The efficiency distributions of RNA edits calculated by MuTect2 were presented for RNA-seq experiments in HEK293T cells that expressed Cas9n, mini ABEmax, ABE8e, ABE8e del153, ABE8s, ABE8s del153 and an sgRNA targeting HEK site 8. Two independent replicates were shown. Each dot represents an edited adenine position in RNA.

(c) Bar plots showing the DNA on-target A-to-G editing efficiencies of Cas9n, ABEmax, del153/dele153*, mini ABEmax, mini del153, ABE8e, ABE8e del153, ABE8s, ABE8s del153 with 6 sgRNAs. The efficiencies for most highly edited three adenines for each sgRNA on-target site within the editing window are reported with two independent replicates, and error bars represent s.d.

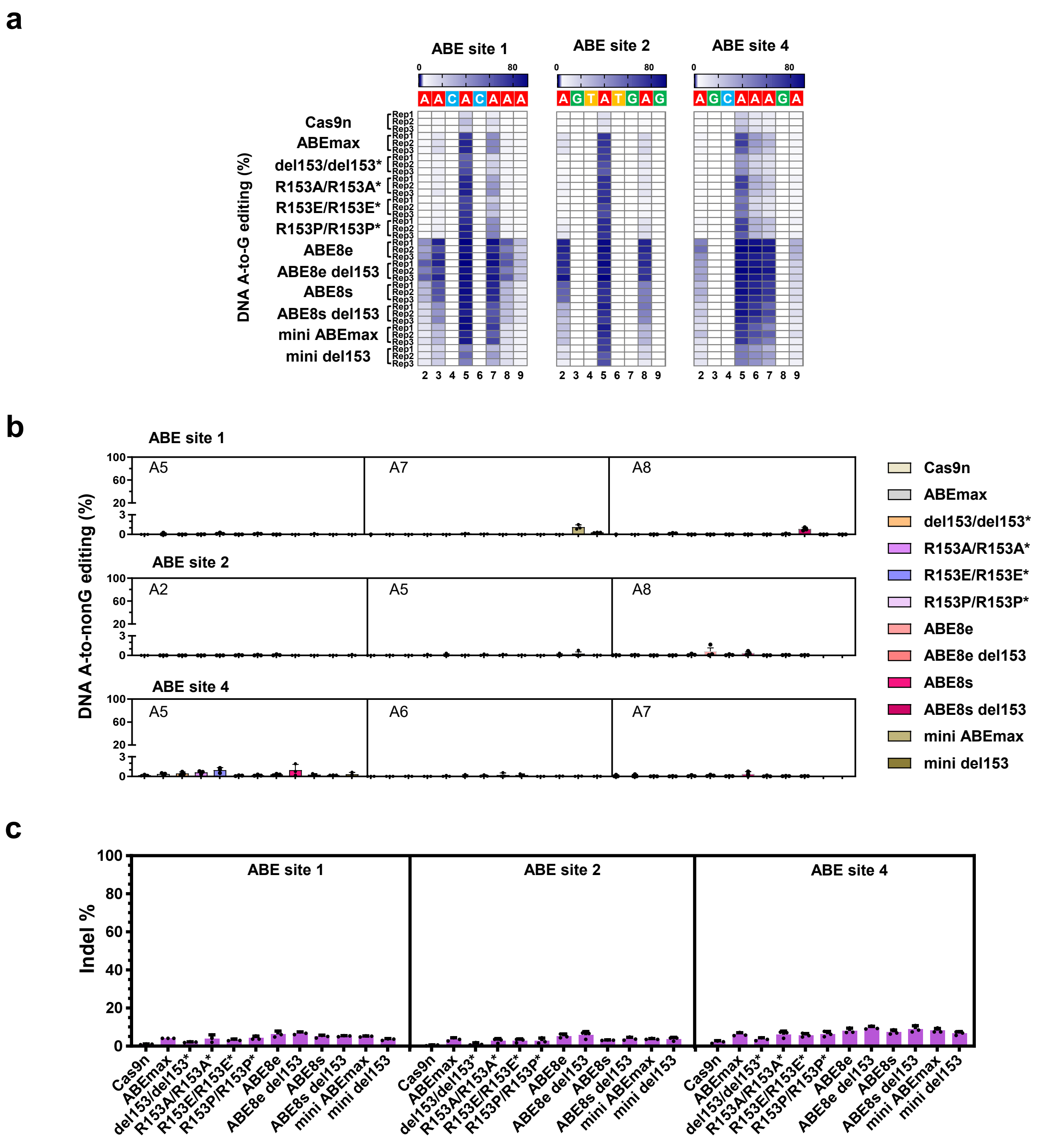

Figure S7. Comparison of engineered ABE variants.

Figure S7. Comparison of engineered ABE variants.

(a) Heat maps showing the on-target DNA A-to-G editing efficiencies of ABEmax and all engineered variants used in the present study for three target sites (ABE site1, 2, and 4). Data are generated from three independent replicates with targeted deep sequencing analysis.

(b-c) Bar plot showing A-to-nonG (A-to-T and A-to-C) editing efficiencies (b) and indel rates (c) induced by indicated base editors. Three independent replicates are presented as mean values ± s.d..

Engineered adenine base editor with minimized RNA off-target activities

SUPPLEMENTARY INFORMATION TABLE

Supplementary Table 1. Oligos used for sgRNA construction in this study

Supplementary Table 2. Primers used for plasmid construction

Supplementary Table 3. Primers used for PCR amplification and sequencing

Supplementary Table 1. Oligos used for sgRNA construction in this study

Name	Sequence
sgHEK293-Site3 up	ACCGGGCCCAGACTGAGCACGTGA
sgHEK293-Site3 down	AAACTCACGTGCTCAGTCTGGGCC
sgABE-Site1 up	ACCGGAACACAAAGCATAGACTGC
sgABE-Site1 down	AAACGCAGTCTATGCTTTGTGTTC
sgABE-Site2 up	ACCGGAGTATGAGGCATAGACTGC
sgABE-Site2 down	AAACGCAGTCTATGCCTCATACTC
sgABE-Site3 up	ACCGGTCAAGAAAGCAGAGACTGC
sgABE-Site3 down	AAACGCAGTCTCTGCTTTCTTGAC
sgABE-Site4 up	ACCGGAGCAAAGAGAATAGACTGT
sgABE-Site4 down	AAACACAGTCTATTCTCTTTGCTC
sgABE-Site5 up	ACCGGATGAGATAATGATGAGTCA
sgABE-Site5 down	AAACTGACTCATCATTATCTCATC
sgABE-Site6 up	ACCGGGATTGACCCAGGCCAGGGC
sgABE-Site6 down	AAACGCCCTGGCCTGGGTCAATCC
sgABE-Site7 up	ACCGGAATACTAAGCATAGACTCC
sgABE-Site7 down	AAACGGAGTCTATGCTTAGTATTC
sgABE-Site8 up	ACCGGTAAACAAAGCATAGACTGA
sgABE-Site8 down	AAACTCAGTCTATGCTTTGTTTAC
sgABE-Site10 up	ACCGGAACATAAAGAATAGAATGA
sgABE-Site10 down	AAACTCATTCTATTCTTTATGTTC
sgABE-Site11 up	ACCGGGACAGGCAGCATAGACTGT
sgABE-Site11 down	AAACACAGTCTATGCTGCCTGTCC
sgABE-Site12 up	ACCGGTAGAAAAAGTATAGACTGC
sgABE-Site12 down	AAACGCAGTCTATACTTTTTCTAC
sgABE-Site14 up	ACCGGGCTAAAGACCATAGACTGT
sgABE-Site14 down	AAACACAGTCTATGGTCTTTAGCC

Supplementary Table 2. Primers used for plasmid construction

Name	Sequence
CMV-NdeI-F	GGCAGTACATCAAGTGTATCATATGCCAAGTACGCCCC
nCas9-BglII-R	GGCCATCTCGTTGCTGAAGATCTCTTGCAGATAGC
TadA-N46A-F	AATAGAGTGATCGGAGAGGGATGGCAAAGGCCAATCGGCCG
TadA-N46A-R	GGCGGCCGATTGGCCTTTGCCATCCCTCTCCGATCACTCTATT
TadA-H57A-F	ACGACCCTACCGCAGCAGCAGAGATCATGGCAC
TadA-H57A-R	GTGCCATGATCTCTGCTGCTGCGGTAGGGTCGT
TadA-R74A-F	GGTCATGCAGAATTACGCGCTGATCGATGCCACC
TadA-R74A-R	ACAGGGTGGCATCGATCAGCGCGTAATTCTGCATGACC
TadA-E85K-F	CCTGTATGTGACACTGAAACCATGCGTGATGTGCGCA
TadA-E85K-R	TGCGCACATCACGCATGGTTTCAGTGTCACATACAGG
TadA*-R98P-F	GCCATGATCCACTCTCCGATCGGCCGCGTGGTGTT
TadA*-R98P-R	AACACCACGCGGCCGATCGGAGAGTGGATCATGGC
TadA-D108A-F	GTGTTCGGAGCACGGGCAGCCAAGACCGGCGCA
TadA-D108A-R	TGCGCCGGTCTTGGCTGCCCGTGCTCCGAACAC
TadA-K110N-F	GGAGCACGGGACGCCAAAACCGGCGCAGCAGGCT

TadA-K110N-R	AGCCTGCTGCGCCGGTTTTGGCGTCCCGTGCTCC
TadA*-N127G-F	CACTACCCCGGCATGGGACACCGCGTCGAAATTA
TadA*-N127G-R	CTCGGTAATTTCGACGCGGTGTCCCATGCCGGGGTAGT
TadA-R153P-F	TCTTTAGAATGCGGCCACAGGAGATCAAGGCC
TadA-R153P-R	GGCCTTGATCTCCTGTGGCCGCATTCTAAAGA
SbfI-HokB-F	AACGCGTGCAGGCCCTGCAGGGCGCCACCATGAAGCACAACCC
SpeI-HokB-R	TTTGGAACCCATGCTTCCGAATTCCCTGGACGTGCAGGC
TadA-R153A-F	TTCTTTAGAATGCGGCGCCAGGAGATCAAGGCC
TadA-R153A-R	GGCCTTGATCTCCTGGCGCCGCATTCTAAAGAA
TadA*-R153A-F	GCCCTGCTGTGCTATTTCTTTCGGATGCCTCCCCAGGTGTTCAATGCT
TadA*-R153A-R	AGCATTGAACACCTGGGGAGGCATCCGAAAGAAATAGCACAGCAGGGCGG
TadA-E59A-F	ACCCTACCGCACACGCAGCAATCATGGCACTGAGGCA
TadA-E59A-R	TGCCTCAGTGCCATGATTGCTGCGTGTGCGGTAGGGT
TadA*-V106W-F	CGCGTGGTGTTTGGCTGGAGGAACGCAAAAACC
TadA*-V106W-R	GGTTTTTGCGTTCCTCCAGCCAAACACCACGCG
TadA-F148A-F	GCCCTGCTGAGCGATGCCTTTAGAATGCGGCCA

TadA-F148A-R	TGGCCGCATTCTAAAGGCATCGCTCAGCAGGGC
TadA*-F148A-F	GCCCTGCTGTGCTATGCCTTTCGGATGCCTAGA
TadA*-F148A-R	TCTAGGCATCCGAAAGGCATAGCACAGCAGGGC
TadA-V82G-F	CGATGCCACCCTGTATGGAACACTGGAGCCATGCGT
TadA-V82G-R	ACGCATGGCTCCAGTGTTCCATACAGGGTGGCATCG
TadA*-V82G-F	ATTGACGCCACCCTGTACGGTACATTCGAGCCTTGC
TadA*-V82G-R	GCAAGGCTCGAATGTACCGTACAGGGTGGCGTCAAT
TadA-K20AR21A-F	GCACTGACCCTGGCAGCGGCGGCATGGGATGAA
TadA-K20AR21A-R	TTCATCCCATGCCGCCGCTGCCAGGGTCAGTGC
TadA*-K20AR21A-F	CATGCCCTGACCCTGGCCATTATAGCACGCGATGAGAGG
TadA*-K20AR21A-R	CCTCTCATCGCGTGCTATAATGGCCAGGGTCAGGGCATG
miniABE-F	AAGAAGCGGAAAGTCTCTGAGGTGGAGTTT
miniABE-R	GAAAACTCCACCTCAGAGACTTTCCGCTTCTT
TadA-del153-F	TTCTTTAGAATGCGGCAGGAGATCAAGGCC

TadA-del153-R	GGCCTTGATCTCCTGCCGCATTCTAAAGAA
TadA*-del153-F	GCCCTGCTGTGCTATTTCTTTCGGATGCCTCAGGTGTTCAATGCTCAG
TadA*-del153-R	CTGAGCATTGAACACCTGTCTAGGCATCCGAAAATAGCACAGCAGGGCGGC
TadA-del148-F	GCCCTGCTGAGCGATTTTAGAATGCGGCCACAG
TadA-del148-R	CTGTGGCCGCATTCTAAAATCGCTCAGCAGGGC
TadA*-del148-F	GCCCTGCTGTGCTATTTTCGGATGCCTAGACAGG
TadA*-del148-R	GTCTAGGCATCCGAAAATAGCACAGCAGGGCGGC
SbfI-HokB-F	AACGCGTGCAGGCCCTGCAGGGCGCCACCATGAAGCACAACCC
SpeI-HokB-R	TTTGGAACCCATGCTTCCGAATTCCCTGGACGTGCAGGC

Supplementary Table 3. Primers used for PCR amplification and deep sequencing

Primer name	Primer sequence
HEK293-Site3 F	GGGAAACGCCCATGCAATTAG
HEK293-Site3 R	CTGCACCGGGATACTGGTTGAC
HEK293 site3 F-1	ATCACGTCTGCTGCAAGTAAGCATGC
HEK293 site3 F-2	CGATGTATCTGCTGCAAGTAAGCATGC
HEK293 site3 F-3	TTAGGCTCTCTGCTGCAAGTAAGCATGC
HEK293 site3 F-4	TGACCACGATCTGCTGCAAGTAAGCATGC
HEK293 site3 F-5	ACAGTGGATGTCTGCTGCAAGTAAGCATGC
HEK293 site3 F-6	GCCAATTCTGCTGCAAGTAAGCATGC
HEK293 site3 F-7	CAGATCATCTGCTGCAAGTAAGCATGC
HEK293 site3 F-8	ACTTGATGTCTGCTGCAAGTAAGCATGC
HEK293 site3 F-9	GATCAGCACTCTGCTGCAAGTAAGCATGC
HEK293 site3 F-10	TAGCTTGTGCTCTGCTGCAAGTAAGCATGC
HEK293 site3 F-11	GGCTACTCTGCTGCAAGTAAGCATGC
HEK293 site3 F-12	CTTGTAATCTGCTGCAAGTAAGCATGC
HEK293 site3 F-13	AGTCAATCTCTGCTGCAAGTAAGCATGC
HEK293 site3 F-14	AGTTCCTTCTGCTGCAAGTAAGCATGC
HEK293 site3 F-15	ATGTCAGCTCTGCTGCAAGTAAGCATGC
HEK293 site3 F-16	CCGTCCATTCTGCTGCAAGTAAGCATGC
HEK293 site3 F-17	GTAGAGCTCTGCTGCAAGTAAGCATGC
HEK293 site3 F-18	GTCCGCATCTGCTGCAAGTAAGCATGC
HEK293 site3 F-19	GTGAAATCTGCTGCAAGTAAGCATGC
HEK293 site3 F-20	GTGGCCTTCTGCTGCAAGTAAGCATGC
HEK293 site3 F-21	GTTTCGTCTGCTGCAAGTAAGCATGC
HEK293 site3 F-22	CGTACGGTCTGCTGCAAGTAAGCATGC
HEK293 site3 F-23	GAGTGGAGTCTGCTGCAAGTAAGCATGC
ABEsite1-F	GGACGTCTGCCCAATATGTA
ABEsite1-R	CAGCCCCATCTGTCAAACTG
ABEsite1-F-1	ATCACGGGACGTCTGCCCAATATGTA
ABEsite1-F-2	CGATGTAGGACGTCTGCCCAATATGTA

ABEsite1-F-3	TTAGGCTCGGACGTCTGCCCAATATGTA
ABEsite1-F-4	TGACCACGAGGACGTCTGCCCAATATGTA
ABEsite1-F-5	ACAGTGGATGGGACGTCTGCCCAATATGTA
ABEsite1-F-6	GCCAATGGACGTCTGCCCAATATGTA
ABEsite1-F-7	CAGATCAGGACGTCTGCCCAATATGTA
ABEsite1-F-8	ACTTGATGGGACGTCTGCCCAATATGTA
ABEsite1-F-9	GATCAGCACGGACGTCTGCCCAATATGTA
ABEsite1-F-10	TAGCTTGTGCGGACGTCTGCCCAATATGTA
ABEsite1-F-11	GGCTACGGACGTCTGCCCAATATGTA
ABEsite1-F-12	CTTGTAAGGACGTCTGCCCAATATGTA
ABEsite1-F-13	AGTCAATCGGACGTCTGCCCAATATGTA
ABEsite1-F-14	AGTTCCTGGACGTCTGCCCAATATGTA
ABEsite1-F-15	ATGTCAGCGGACGTCTGCCCAATATGTA
ABEsite1-F-16	CCGTCCATGGACGTCTGCCCAATATGTA
ABEsite1-F-17	GTAGAGCGGACGTCTGCCCAATATGTA
ABEsite1-F-18	GTCCGCAGGACGTCTGCCCAATATGTA
ABEsite1-F-19	GTGAAAGGACGTCTGCCCAATATGTA
ABEsite1-F-20	GTGGCCTGGACGTCTGCCCAATATGTA
ABEsite1-F-21	GTTTCGGGACGTCTGCCCAATATGTA
ABEsite1-F-22	CGTACGGGGACGTCTGCCCAATATGTA
ABEsite1-F-23	GAGTGGAGGGACGTCTGCCCAATATGTA
ABEsite1-F-24	GGTAGCAGGACGTCTGCCCAATATGTA
ABEsite2-F	CCTGAGATACAGTCACGAGGT
ABEsite2-R	TCCTGAAATGCTGTGCGTGT
ABEsite2-F-1	ATCACGCCTGAGATACAGTCACGAGGT
ABEsite2-F-2	CGATGTACCTGAGATACAGTCACGAGGT
ABEsite2-F-3	TTAGGCTCCCTGAGATACAGTCACGAGGT
ABEsite2-F-4	TGACCACGACCTGAGATACAGTCACGAGGT
ABEsite2-F-5	ACAGTGGATGCCTGAGATACAGTCACGAGGT
ABEsite2-F-6	GCCAATCCTGAGATACAGTCACGAGGT
ABEsite2-F-7	CAGATCACCTGAGATACAGTCACGAGGT
ABEsite2-F-8	ACTTGATGCCTGAGATACAGTCACGAGGT

ABEsite2-F-9	GATCAGCACCCTGAGATACAGTCACGAGGT
ABEsite2-F-10	TAGCTTGTGCCCTGAGATACAGTCACGAGGT
ABEsite2-F-11	GGCTACCCTGAGATACAGTCACGAGGT
ABEsite2-F-12	CTTGTAACCTGAGATACAGTCACGAGGT
ABEsite2-F-13	AGTCAATCCCTGAGATACAGTCACGAGGT
ABEsite2-F-14	AGTTCCTCCTGAGATACAGTCACGAGGT
ABEsite2-F-15	ATGTCAGCCCTGAGATACAGTCACGAGGT
ABEsite2-F-16	CCGTCCATCCTGAGATACAGTCACGAGGT
ABEsite2-F-17	GTAGAGCCCTGAGATACAGTCACGAGGT
ABEsite2-F-18	GTCCGCACCTGAGATACAGTCACGAGGT
ABEsite2-F-19	GTGAAACCTGAGATACAGTCACGAGGT
ABEsite2-F-20	GTGGCCTCCTGAGATACAGTCACGAGGT
ABEsite2-F-21	GTTTCGCCTGAGATACAGTCACGAGGT
ABEsite2-F-22	CGTACGGCCTGAGATACAGTCACGAGGT
ABEsite2-F-23	GAGTGGAGCCTGAGATACAGTCACGAGGT
ABEsite2-F-24	GGTAGCACCTGAGATACAGTCACGAGGT
ABEsite3-F	GCTTTTCACCGACTGCACAG
ABEsite3-R	CCGACAGCCAGTGGTTAAGT
ABEsite3-F-1	ATCACGGCTTTTCACCGACTGCACAG
ABEsite3-F-2	CGATGTAGCTTTTCACCGACTGCACAG
ABEsite3-F-3	TTAGGCTCGCTTTTCACCGACTGCACAG
ABEsite3-F-4	TGACCACGAGCTTTTCACCGACTGCACAG
ABEsite3-F-5	ACAGTGGATGGCTTTTCACCGACTGCACAG
ABEsite3-F-6	GCCAATGCTTTTCACCGACTGCACAG
ABEsite3-F-7	CAGATCAGCTTTTCACCGACTGCACAG
ABEsite3-F-8	ACTTGATGGCTTTTCACCGACTGCACAG
ABEsite3-F-9	GATCAGCACGCTTTTCACCGACTGCACAG
ABEsite3-F-10	TAGCTTGTGCGCTTTTCACCGACTGCACAG
ABEsite3-F-11	GGCTACGCTTTTCACCGACTGCACAG
ABEsite3-F-12	CTTGTAAGCTTTTCACCGACTGCACAG
ABEsite3-F-13	AGTCAATCGCTTTTCACCGACTGCACAG
ABEsite3-F-14	AGTTCCTGCTTTTCACCGACTGCACAG

ABEsite3-F-15	ATGTCAGCGCTTTTCACCGACTGCACAG
ABEsite3-F-16	CCGTCCATGCTTTTCACCGACTGCACAG
ABEsite3-F-17	GTAGAGCGCTTTTCACCGACTGCACAG
ABEsite3-F-18	GTCCGCAGCTTTTCACCGACTGCACAG
ABEsite3-F-19	GTGAAAGCTTTTCACCGACTGCACAG
ABEsite3-F-20	GTGGCCTGCTTTTCACCGACTGCACAG
ABEsite3-F-21	GTTTCGGCTTTTCACCGACTGCACAG
ABEsite3-F-22	CGTACGGGCTTTTCACCGACTGCACAG
ABEsite3-F-23	GAGTGGAGGCTTTTCACCGACTGCACAG
ABEsite3-F-24	GGTAGCAGCTTTTCACCGACTGCACAG
ABEsite4-F	TCCTTGCACTGAGACCGTGAA
ABEsite4-R	CTGCACCTAGCCTCCATGTC
ABEsite4-F-1	ATCACGTCCTTGCACTGAGACCGTGAA
ABEsite4-F-2	CGATGTATCCTTGCACTGAGACCGTGAA
ABEsite4-F-3	TTAGGCTCTCCTTGCACTGAGACCGTGAA
ABEsite5-F	GTCTGAGGTCACACAGTGGG
ABEsite5-R	TCTGAGAGCAGGGACCACATC
ABEsite5-F-1	ATCACGGTCTGAGGTCACACAGTGGG
ABEsite5-F-2	CGATGTAGTCTGAGGTCACACAGTGGG
ABEsite5-F-3	TTAGGCTCGTCTGAGGTCACACAGTGGG
ABEsite6-F	GGGAAACGCCCATGCAATTA
ABEsite6-R	GTCAACCAGTATCCCGGTGC
ABEsite6-F-1	ATCACGGGGAAACGCCCATGCAATTA
ABEsite6-F-2	CGATGTAGGGAAACGCCCATGCAATTA
ABEsite6-F-3	TTAGGCTCGGGAAACGCCCATGCAATTA
ABEsite7-F	GATGCCCTCCATCTTCTCCG
ABEsite7-R	TAGGTTTGCATAGACCTGCCC
ABEsite7-F-1	ATCACGGATGCCCTCCATCTTCTCCG
ABEsite7-F-2	CGATGTAGATGCCCTCCATCTTCTCCG
ABEsite7-F-3	TTAGGCTCGATGCCCTCCATCTTCTCCG
ABEsite8-F	GCAAGGCTGCTAGAAGTTTCA
ABEsite8-R	GCAGAAGGAATAACAGTGCCC

ABEsite8-F-1	ATCACGGCAAGGCTGCTAGAAGTTTCA
ABEsite8-F-2	CGATGTAGCAAGGCTGCTAGAAGTTTCA
ABEsite8-F-3	TTAGGCTCGCAAGGCTGCTAGAAGTTTCA
ABEsite9-F	CCTCCATCTTCCTACACGCC
ABEsite9-R	GAACAGGCAGCGTATTGCTT
ABEsite9-F-1	ATCACGCCTCCATCTTCCTACACGCC
ABEsite9-F-2	CGATGTACCTCCATCTTCCTACACGCC
ABEsite9-F-3	TTAGGCTCCCTCCATCTTCCTACACGCC
ABEsite10-F	TCCACCTCCCCACTTCTCTT
ABEsite10-R	GGTGAAATGAGCAAGGCACA
ABEsite10-F-1	ATCACGTCCACCTCCCCACTTCTCTT
ABEsite10F--2	CGATGTATCCACCTCCCCACTTCTCTT
ABEsite10-F-3	TTAGGCTCTCCACCTCCCCACTTCTCTT
ABEsite11-F	AACCACCTGCAGAGGACTACT
ABEsite11-R	GAAGGCCTAGAAAGAGACAGC
ABEsite11-F-1	ATCACGAACCACCTGCAGAGGACTACT
ABEsite11-F-2	CGATGTAAACCACCTGCAGAGGACTACT
ABEsite11-F-3	TTAGGCTCAACCACCTGCAGAGGACTACT
ABEsite12-F	TGGTGATTATGGTTACACAGCG
ABEsite12-R	ACCCATGTGCCTGACATAGG
ABEsite12-F-1	ATCACGTGGTGATTATGGTTACACAGCG
ABEsite12-F-2	CGATGTATGGTGATTATGGTTACACAGCG
ABEsite12-F-3	TTAGGCTCTGGTGATTATGGTTACACAGCG
ABEsite13-F	TATCAAACACTGGGTCAT
ABEsite13-R	CAAGCCAAATTCAACAAC
ABEsite13-F-1	ATCACGTATCAAACACTGGGTCAT
ABEsite13-F-2	CGATGTATATCAAACACTGGGTCAT
ABEsite13-F-3	TTAGGCTCTATCAAACACTGGGTCAT
ABEsite14-F	GGATTACAGCCTGAGCCA
ABEsite14-R	CCAACTGGTAACCCACAAA
ABEsite14-F-1	ATCACGGGATTACAGCCTGAGCCA
ABEsite14-F-2	CGATGTAGGATTACAGCCTGAGCCA

ABEsite14-F-3	TTAGGCTCGGATTACAGCCTGAGCCA
ABE-site16-F	TCCTGAGGTCTAGGAACCCG
ABE-site16-R	GGGAGGTGGAGAGAGGATGT
HokB F-1	ATCACGATGAAGCACAACCCT
HokB F-2	CGATGTAATGAAGCACAACCCT
HokB F-3	TTAGGCTCATGAAGCACAACCCT
HokB F-4	TGACCACGAATGAAGCACAACCCT
HokB F-5	ACAGTGGATGATGAAGCACAACCCT
HokB F-6	GCCAATATGAAGCACAACCCT
HokB F-7	CAGATCAATGAAGCACAACCCT
HokB F-8	ACTTGATGATGAAGCACAACCCT
HokB F-9	GATCAGCACATGAAGCACAACCCT
HokB F-10	TAGCTTGTGCATGAAGCACAACCCT
HokB F-11	GGCTACATGAAGCACAACCCT
HokB F-12	CTTGTAAATGAAGCACAACCCT
HokB F-13	AGTCAATCATGAAGCACAACCCT
HokB F-14	AGTTCCTATGAAGCACAACCCT
HokB F-15	ATGTCAGCATGAAGCACAACCCT
HokB F-16	CCGTCCATATGAAGCACAACCCT
HokB F-17	GTAGAGCATGAAGCACAACCCT
HokB F-18	GTCCGCAATGAAGCACAACCCT
HokB R	GGCCTGCACGTCCAGGT
ABE-MU-OFT-1-F	CGACCGCATCTACGACCTCAAC
ABE-MU-OFT-1-R	TGAGCTCCTCCTCGGTGACTG
ABE-MU-OFT-2-F	CCATCACAGGGTCCAGCGAG
ABE-MU-OFT-2-R	TACACAGGTCTCAGCCGAAGC
ABE-MU-OFT-3-F	ATTCATACCGGGGAGAAGCCC
ABE-MU-OFT-3-R	TCGGAAGGCGTTCCTCCTGTG
ABE-MU-OFT-4-F	GGGCAGCATCCACACGGTTAG
ABE-MU-OFT-4-R	CCCCTTCATCCTCTCTGAGCTTG
ABE-MU-OFT-5-F	TTCCCAAGCGGCTGCCGAAG
ABE-MU-OFT-5-R	GGCAGCAGGAACCACCATCCG

ABE-MU-OFT-6-F	CCTCCTCTGAAGTCACGGAGC
ABE-MU-OFT-6-R	CAGGTCATCCTCTTTGTGGTCAC
ABE-MU-OFT-7-F	CTCTTCCGGTTCTAGGCACTTCG
ABE-MU-OFT-7-R	TGGGATCTTGGGCTTAACCTCC
ABE-MU-OFT-8-F	ATGACCACCCCAGGAAAAGAG
ABE-MU-OFT-8-R	TGGTGCCATCTCCATGTTAC
