# Supplementary material for: Structure-guided engineering of adenine base editor with minimized RNA off-targeting activity
Source: Nat Commun. 2021 Apr 16;12:2287. doi: 10.1038/s41467-021-22519-z (PMC8052359; doi:10.1038/s41467-021-22519-z)
Supplement: Supplementary file 2 — Reporting Summary [file 41467_2021_22519_MOESM2_ESM.pdf]

nature research

Corresponding author(s): Yunbo Qiao
Last updated by author(s): Mar 9, 2021

Reporting Summary

Nature Research wishes to improve the reproducibility of the work that we publish. This form provides structure for consistency and transparency in reporting. For further information on Nature Research policies, see our Editorial Policies and the Editorial Policy Checklist.

Statistics

For all statistical analyses, confirm that the following items are present in the figure legend, table legend, main text, or Methods section.

n/a	Confirmed	
[ ]	[x]	The exact sample size (*n*) for each experimental group/condition, given as a discrete number and unit of measurement
[ ]	[x]	A statement on whether measurements were taken from distinct samples or whether the same sample was measured repeatedly
[ ]	[x]	The statistical test(s) used AND whether they are one- or two-sided<br>*Only common tests should be described solely by name; describe more complex techniques in the Methods section.*
[ ]	[x]	A description of all covariates tested
[ ]	[x]	A description of any assumptions or corrections, such as tests of normality and adjustment for multiple comparisons
[ ]	[x]	A full description of the statistical parameters including central tendency (e.g. means) or other basic estimates (e.g. regression coefficient) AND variation (e.g. standard deviation) or associated estimates of uncertainty (e.g. confidence intervals)
[x]	[ ]	For null hypothesis testing, the test statistic (e.g. *F*, *t*, *r*) with confidence intervals, effect sizes, degrees of freedom and *P* value noted<br>*Give P values as exact values whenever suitable.*
[x]	[ ]	For Bayesian analysis, information on the choice of priors and Markov chain Monte Carlo settings
[ ]	[x]	For hierarchical and complex designs, identification of the appropriate level for tests and full reporting of outcomes
[x]	[ ]	Estimates of effect sizes (e.g. Cohen's *d*, Pearson's *r*), indicating how they were calculated

Our web collection on statistics for biologists contains articles on many of the points above.

Software and code

Policy information about availability of computer code

Data collection	The reads were mapped to the human reference genome (hg38) by STAR software (Version 2.5.1), annotation from GENCODE version v30 was used. After removing duplication, variants were identified by GATK (Version 4.1.8.1; MuTect2 and HaplotypeCaller) .
Data analysis	BWA (V0.7.16) and Samtools (V1.9) was employed for mapping the pair-end reads to human reference genome (hg38). The adapter pair of the pair-end reads were removed using AdapterRemoval version 2.2.2, and pair-end read alignments of 11 bp or more bases were combined into a single consensus read. All processed reads were then mapped to the target sequences using the BWA-MEM algorithm (BWA V0.7.16). For each site, the mutation rate was calculated using bam-readcount with parameters -q 20 -b 30. Indels were calculated based on reads containing at least 1 inserted or deleted nucleotide in protospacer. Indel frequency was calculated as the number of indel-containing reads/ total mapped reads.<br>For MuTect2 method, variants were filtered with FilterMutectCalls. For HaplotypeCaller method, variants were first filtered with QD (Quality by Depth) < 2, then all variants were verified and quantified by bam-readcount with parameters -q 20 -b 30. The depth for a given edit should be at least 10x and these edits were required to have at least 99% of reads supporting the reference allele in the wild-type samples. Finally, only A-to-G edits in transcribed strand were considered for downstream analysis. Motif or sequence logo was analyzed by WebLogo (v3.6.0) for RNA edits.

For manuscripts utilizing custom algorithms or software that are central to the research but not yet described in published literature, software must be made available to editors and reviewers. We strongly encourage code deposition in a community repository (e.g. GitHub). See the Nature Research guidelines for submitting code & software for further information.

Data

Policy information about availability of data

All manuscripts must include a data availability statement. This statement should provide the following information, where applicable:

- Accession codes, unique identifiers, or web links for publicly available datasets
- A list of figures that have associated raw data
- A description of any restrictions on data availability

A structural model for TadA-RNA complex was generated using coordinates from PDB ID 2B3J (weblink: http://www.rcsb.org/structure/2b3j) by PyMol (The PyMOL Molecular Graphics System, Version 1.9 Schrödinger, LLC.).
All sequencing data was provided in SRA (accession number: PRJNA660634 [https://www.ncbi.nlm.nih.gov/bioproject/?term=prjna660634]). All other results in the present study are available upon request.

Field-specific reporting

Please select the one below that is the best fit for your research. If you are not sure, read the appropriate sections before making your selection.

☒ Life sciences ☐ Behavioural & social sciences ☐ Ecological, evolutionary & environmental sciences

For a reference copy of the document with all sections, see nature.com/documents/nr-reporting-summary-flat.pdf

Life sciences study design

All studies must disclose on these points even when the disclosure is negative.

Sample size	No statistical method was used to predetermine sample size. Sample sizes were determined according to requirements for each comparisons in order to unravel meaningful conclusions and according to the throughput and dimensionality of experimental measurements across different group of experiment with multiple plasmids. Especially, it included positive and negative controls regarding RNA off-targeting analysis for each experiment.
Data exclusions	Low transfection efficiency for individual groups (much lower than other comparing groups) were excluded in RNA-seq experiments.
Replication	All data were replicated using two or three biologically independent experiments. To assure the reproducibility of our findings regarding the DNA editing efficiency, all PCR fragments were subjected to Sanger sequencing or targeted deep sequencing, or both. All results were confirmed in replications with consistent conclusions in the present study.
Randomization	Plasmids and comparisons were not randomized in multi-groups, and the assigned groups were sure for each experiment. For each group of experiments, a same batch of cells were plated into different wells or dishes, and were transfected with different plasmids as required for comparisons.
Blinding	Blinding was not performed for this study, because the investigators had no prior assumption/bias regarding the expected outcomes, and the findings were summarized from an unbiased results.

Reporting for specific materials, systems and methods

We require information from authors about some types of materials, experimental systems and methods used in many studies. Here, indicate whether each material, system or method listed is relevant to your study. If you are not sure if a list item applies to your research, read the appropriate section before selecting a response.

Materials & experimental systems

n/a	Involved in the study	
☒	☐	Antibodies
☐	☒	Eukaryotic cell lines
☒	☐	Palaeontology and archaeology
☒	☐	Animals and other organisms
☒	☐	Human research participants
☒	☐	Clinical data
☒	☐	Dual use research of concern

Methods

n/a	Involved in the study	
☒	☐	ChIP-seq
☐	☒	Flow cytometry
☒	☐	MRI-based neuroimaging

Eukaryotic cell lines

Policy information about cell lines

Cell line source(s)	HEK293T cells, U2OS cells
Authentication	U2OS cell was authenticated by using RNA-seq sequences to compare with COSMIC database with GATK (Version 4.1.8.1). HEK293T cell was not authenticated.
Mycoplasma contamination	We confirmed that there is no mycoplasma contamination for used cells.
Commonly misidentified lines (See ICLAC register)	None

Flow Cytometry

Plots

Confirm that:

- [x] The axis labels state the marker and fluorochrome used (e.g. CD4-FITC).
- [x] The axis scales are clearly visible. Include numbers along axes only for bottom left plot of group (a 'group' is an analysis of identical markers).
- [x] All plots are contour plots with outliers or pseudocolor plots.
- [x] A numerical value for number of cells or percentage (with statistics) is provided.

Methodology

Sample preparation	Tranfected cells were disassociated into single cells, washed with PBS, and subjected to collection of top 15% GFP positive cells
Instrument	FACSAriaIII
Software	FACSDiva was used for managing the instrument.
Cell population abundance	20-30 million cells were collected for RNA extraction and RNA-seq analysis.
Gating strategy	Top 15% GFP positive cells were collected, and global transfection efficiency was comparable between different groups.

- [x] Tick this box to confirm that a figure exemplifying the gating strategy is provided in the Supplementary Information.
